# Electrophysiological evidence of the time course of attentional bias in non-patients reporting symptoms of depression with and without co-occurring anxiety

**DOI:** 10.3389/fpsyg.2014.00301

**Published:** 2014-04-09

**Authors:** Sarah M. Sass, Wendy Heller, Joscelyn E. Fisher, Rebecca L. Silton, Jennifer L. Stewart, Laura D. Crocker, J. Christopher Edgar, Katherine J. Mimnaugh, Gregory A. Miller

**Affiliations:** ^1^Department of Psychology, University of Texas at TylerTyler, TX, USA; ^2^Department of Psychology, University of Illinois at Urbana-ChampaignChampaign, IL, USA; ^3^Department of Psychiatry, Uniformed Services University of the Health SciencesBethesda, MD, USA; ^4^Department of Psychology, Loyola University ChicagoChicago, IL, USA; ^5^Department of Psychiatry, University of California at San DiegoSan Diego, CA, USA; ^6^Department of Radiology, University of PennsylvaniaPhiladelphia, PA, USA; ^7^Department of Psychology, University of California at Los AngelesLos Angeles, CA, USA; ^8^Department of Psychiatry and Biobehavioral Sciences, University of California at Los AngelesLos Angeles, CA, USA

**Keywords:** attentional bias, anxiety, depression, emotion, event-related brain potentials

## Abstract

Anxiety is characterized by attentional biases to threat, but findings are inconsistent for depression. To address this inconsistency, the present study systematically assessed the role of co-occurring anxiety in attentional bias in depression. In addition, the role of emotional valence, arousal, and gender was explored. Ninety-two non-patients completed the Penn State Worry Questionnaire (Meyer et al., 1990; Molina and Borkovec, 1994) and portions of the Mood and Anxiety Symptom Questionnaire (Watson et al., 1995a,1995b). Individuals reporting high levels of depression and low levels of anxiety (depression only), high levels of depression and anxiety (combined), or low levels of both (control) completed an emotion-word Stroop task during event-related brain potential recording. Pleasant and unpleasant words were matched on emotional arousal level. An attentional bias was not evident in the depression-only group. Women in the combined group had larger N200 amplitude for pleasant than unpleasant stimuli, and the combined group as a whole had larger right-lateralized P300 amplitude for pleasant than unpleasant stimuli, consistent with an early and later attentional bias that is specific to unpleasant valence in the combined group. Men in the control group had larger N200 amplitude for pleasant than unpleasant stimuli, consistent with an early attentional bias that is specific to pleasant valence. The present study indicates that the nature and time course of attention prompted by emotional valence and not arousal differentiates depression with and without anxiety, with some evidence of gender moderating early effects. Overall, results suggest that co-occurring anxiety is more important than previously acknowledged in demonstrating evidence of attentional biases in depression.

## INTRODUCTION

An impressive body of research has demonstrated that depression and anxiety are characterized by cognitive biases, including attentional bias or preferential attentional processing of unpleasant or threatening information. Attentional bias has been argued to contribute to the etiology and maintenance of anxiety and depression (e.g., Beck, 1976; Beck et al., 2005; Bar-Haim et al., 2007; Levin et al., 2007; Gotlib and Joormann, 2010). Whereas anxiety has been consistently associated with attentional biases to threatening or emotionally arousing stimuli (Williams et al., 1996; McNally, 1998; Becker et al., 2001; Fox et al., 2002), evidence of attentional biases in depression has been mixed (Mogg and Bradley, 2005; Gotlib and Joormann, 2010), with some studies finding preferential processing of unpleasant stimuli (Gotlib and Cane, 1987; Bradley et al., 1997), others insufficient attention to pleasant stimuli (Gotlib et al., 1988; McCabe and Gotlib, 1995; Gilboa and Gotlib, 1997; McCabe et al., 2000), and others a lack of differentiation between pleasant and unpleasant stimuli (e.g., McCabe and Gotlib, 1995; Deldin et al., 2001).

Along with inconsistent evidence regarding the nature of attentional biases in depression, evidence regarding the time course of bias has also been inconsistent. Understanding the time course of attentional processing is critical in elucidating the degree to which early registration and vigilance, relying primarily on early sensory processing involving brain areas such as visual cortex and amygdala, or later, more elaborative attentional and cognitive-control mechanisms, relying primarily on cortical and prefrontal regions (for reviews, see Bishop, 2007; Gotlib and Joormann, 2010), are involved in abnormal attention to emotional information. Some evidence exists for an early attentional bias in favor of unpleasant information in depression, especially from electrophysiological studies (e.g., Williams et al., 2007; Jaworska et al., 2010). Evidence of a later bias in favor of unpleasant information in depression also exists (e.g., Gotlib and Joormann, 2010), supporting the hypothesis that later attentional processes (such as difficulty disengaging from or inhibiting unpleasant information) are involved in attentional biases in depression (e.g., Leyman et al., 2007; Joormann and D’Avanzato, 2010).

Depression and anxiety are frequently co-occurring and share considerable conceptual and measurement overlap (Clark and Watson, 1991; Heller et al., 1998; Keller et al., 2000; Nitschke et al., 2001; Watson, 2009). Surprisingly few studies account for anxiety in attentional bias investigations of depression (Bar-Haim et al., 2007), despite the potential for co-occurring anxiety to affect the nature or timing of bias in depression. A large literature documents evidence of both early (e.g., Williams et al., 1996; Bar-Haim et al., 2007; Eysenck et al., 2007; Li et al., 2007; Sass et al., 2010) and later (e.g., Fox et al., 2002; Li et al., 2007) attentional biases in anxiety, with evidence of attention being captured quickly by threatening stimuli (e.g., Williams et al., 1996), difficulty disengaging from threatening stimuli once attended (e.g., Fox et al., 2002), initial engagement with threatening stimuli followed by avoidance (e.g., Mogg et al., 2004), and preferential engagement with both pleasant and threatening compared to neutral information (e.g., Martin et al., 1991; Sass et al., 2010). In samples with depression and co-occurring anxiety, evidence of attentional bias is sometimes found (e.g., Rossignol et al., 2008; Markela-Larenc et al., 2011) and sometimes not (Bradley et al., 1995).

Co-occurring anxiety can also affect patterns of brain activity in regions implementing attentional control (e.g., Heller, 1990, 1993; Heller et al., 1995; Heller and Nitschke, 1997; Keller et al., 2000). Although anxiety is a broad construct that is often treated monolithically, anxious apprehension (worry) and anxious arousal (panic or sympathetic arousal), are accompanied by distinct patterns of abnormal activity in brain regions implementing attentional control (e.g., Heller et al., 1997; Heller and Nitschke, 1998; Engels et al., 2007, 2010) and are associated with a dissociable time course of attentional bias to emotionally arousing stimuli (Sass et al., 2010). In order to examine the neural mechanisms involved in the time course of attentional disruption in depression with and without co-occurring anxiety, it is important to investigate these dimensions of anxiety in conjunction with depression.

Different patterns of attentional biases depending on depression and anxiety co-occurrence could have substantial implications for treatment. Unique cognitive characteristics of depression with and without anxiety (and potential targets for intervention) could be associated with different neural mechanisms. Co-occurring anxiety can affect patterns of brain activity that are related to attentional processing (e.g., Heller, 1990, 1993; Heller et al., 1995; Heller and Nitschke, 1997; Keller et al., 2000; Engels et al., 2010). Resting EEG, functional magnetic resonance imaging (fMRI), and event-related brain potential (ERP) studies provide evidence of lateralization patterns in depression with less right than left activity over parieto-temporal regions (e.g., Deldin et al., 2000; Engels et al., 2010; Stewart et al., 2011). These posterior brain regions are associated with vigilance and autonomic arousal, and less activity in these areas in depressive states is presumably due to less arousal characterized by symptoms such as anhedonia (e.g., Heller and Nitschke, 1998; Engels et al., 2010). For example, ERP studies demonstrate evidence of reduced right parietal N200 amplitude (Deldin et al., 2000) and P300 amplitude (Sumich et al., 2006) in depressed individuals. Conversely, depression with co-occurring anxious apprehension has been associated with greater right inferior occipital cortex fMRI activity and co-occurring anxious arousal with greater right inferior temporal gyrus fMRI activity in response to unpleasant vs. neutral information in the context of an emotion-word Stroop task (Engels et al., 2010). Examining lateralized neural mechanisms reflecting the time course of processing of emotional stimuli may provide critical insights in understanding biased processing of emotional stimuli in depression with and without co-occurring anxiety.

In addition to co-occurring anxiety, emotional valence and emotional arousal are important to systematically investigate in attentional biases in depression. Pleasant stimuli are inconsistently included in attentional bias studies and when they are included, are not consistently matched to unpleasant stimuli on emotional arousal level (e.g., see Mogg and Bradley, 2005, **Table 1**; Williams et al., 1996). It is possible that a general emotional arousal confound contributes to variance in findings. That is, it may be the high emotional arousal value of unpleasant stimuli and not unpleasant valence *per se* that drives attentional biases in depression. In order to assess this issue, the present study matched unpleasant and pleasant stimuli on emotional arousal level.

**Table 1:** Means and standard deviations for questionnaire scores used to form groups.

	*Group Questionnaire Scores*		
	PSWQ	MASQ-AA	MASQ-AD
**Group**
Depression-only	36 (9.4)	22 (2.5)	25 (2.8)
Combined	71 (5.4)	71 (5.4)	27 (3.9)
Control	38 (8.6)	20 (2.2)	13 (2.4)

*PSWQ refers to the Penn State Worry Questionnaire. MASQ-AA and MASQ-AD refer to the anxious arousal and anhedonic depression subscales of the Mood and Symptom Questionnaire, respectively.*

Gender is also important to investigate systematically in attentional bias research. Women are estimated to suffer from depression and anxiety twice as often as men (Weissman et al., 1996; Nolen-Hoeksema, 2001; Craske, 2003). Several studies indicate that gender moderates emotional information processing in depressed (e.g., Wright et al., 2009) and anxious (e.g., Sass et al., 2010) participants. For example, depressed women took longer to categorize negative faces than did control women, whereas depressed men performed no differently than control men (Wright et al., 2009). Failing to include gender may further contribute to inconsistency regarding the nature and timing of attentional biases in depression and may unnecessarily limit understanding of how these biases contribute to and maintain depression. Gender also moderates processing of emotional stimuli in control participants, with women sometimes showing evidence of preferential processing of unpleasant stimuli (e.g., Lang et al., 1998), and men tending to show the opposite pattern, prioritizing pleasant information (Lang et al., 1998; Bradley and Lang, 2007). A more comprehensive understanding of the nature and time course of the processing of emotional stimuli in control participants may provide useful information regarding the higher prevalence rates of depression and anxiety in women.

In examining attention to emotional stimuli in depression and anxiety, many studies have used an emotion-word variant of the Stroop task. Distracter word content is unpleasant (“assault”), neutral (“cabinet”), or pleasant (“festive”), and participants are asked to ignore the content or meaning of the word while responding to the color of the word. A recent meta-analysis indicated that clinically depressed individuals show slower color naming in the emotion-word Stroop task for unpleasant than for neutral words, consistent with biased processing of unpleasant information (Epp et al., 2012). Similarly, in anxiety, a large literature demonstrates that color naming is slowed in anxious participants when the distracter word is unpleasant or threatening, with larger effects in individuals diagnosed with anxiety disorders and smaller or inconsistent effects in individuals with self-reported trait or state anxiety (e.g., Williams et al., 1996; Koven et al., 2003; Bar-Haim et al., 2007). Reaction time (RT) alone may not be a precise indicator of attentional bias given that delayed RT can be interpreted as avoidance instead of heightened attention toward negative stimuli (e.g., De Raedt and Koster, 2010). In contrast, ERP methodology offers high temporal resolution that can differentiate early sensory from later more elaborative processing prior to response selection and execution. In general, early sensory processing occurs prior to 300 ms (e.g., Luck et al., 2000), and later conflict detection processes occur beginning approximately 300–600 ms (e.g., Donchin and Coles, 1988; Coles et al., 2000; West, 2003).

The present study focused on P100 and posterior visual N200 amplitude as indices of earlier, more automatic stimulus processing, and P300 (sometimes called P3b, late positive potential (LPP), or late positive complex (LPC) as an index of later, more elaborative stimulus processing. P100 amplitude peaks approximately 100 ms after stimulus onset and grows larger as more extrastriate cortex resources are devoted to processing stimuli (Luck et al., 2000). P100 was larger for sad than for joyful facial expressions (Jaworska et al., 2010) and smaller for positive words in depressed than in control participants (Dai and Feng, 2011). In anxious participants in the emotion-word Stroop task, P100 was larger for unpleasant than neutral stimuli (e.g., Li et al., 2007) and for unpleasant and pleasant than neutral stimuli (Sass et al., 2010).

Posterior visual N200 (what is sometimes called N100) immediately follows P100 over occipito-parietal sensors (e.g., Allison et al., 2002; Ruz and Nobre, 2008; Sass et al., 2010), but peaks later (approximately 200 ms) than classical N100 elicited in visual attention tasks (e.g., Gonzalez et al., 1994; Anllo-Vento and Hillyard, 1996), especially those using short intertrial intervals (ITIs). N200 likely originates in extrastriate cortex and is maximal over bilateral occipital-posterior regions [sometimes called early posterior negativity (EPN); e.g., Weber et al., 2009]. In depression, smaller N200 for happy than sad faces (e.g., Deldin et al., 2000) or no modulation of N200 amplitude in response to emotional stimuli (e.g., Kayser et al., 2000) has been found. In anxiety, larger N200 amplitude has been associated with processing emotionally arousing than neutral stimuli in the context of an emotion-word Stroop task (Sass et al., 2010). Taken together, P100 and N200 amplitude results indicate stronger evidence for an early bias in anxiety but mixed evidence for an early bias in depression, mirroring the behavioral literature. The posterior N200 component in the present study can be distinguished from a fronto-central N200 component that is thought to be associated with effortful processing (such as inhibition and conflict monitoring; e.g., Donkers and van Boxtel, 2004), and which typically peaks later in time (between 200 and 500 ms; e.g., Thomas et al., 2007; Enriquez-Geppert et al., 2010).

P300 amplitude is associated with context updating and event categorization processes (e.g., Donchin and Coles, 1988) as well as increased resource engagement (e.g., Yee and Miller, 1994). P300 amplitude is often modulated by emotional arousal, with larger amplitude for emotionally arousing than neutral stimuli interpreted as reflecting more attentional resources devoted to processing these stimuli (e.g., Schupp et al., 2004; Fischler and Bradley, 2006; Thomas et al., 2007; Li et al., 2007; Franken et al., 2009; Sass et al., 2010). In anxiety, P300 amplitude has been larger for unpleasant than neutral words (Li et al., 2007) and for emotionally arousing (pleasant and unpleasant) than neutral words (no difference between anxious and control participants, Sass et al., 2010) in the context of an emotion-word Stroop task. In comorbid anxiety and depression, no P300 effects were found in a visual oddball task including happy, sad, and neutral faces (Rossignol et al., 2008). Thus, inconsistent P300 amplitude evidence exists for a later attentional resource allocation bias for unpleasant or emotionally arousing stimuli in both depression and anxiety.

In order to address questions concerning the role of emotional valence, emotional arousal, co-occurring anxiety, and gender on the nature and timing of attentional biases in depression, the present study examined ERPs in three groups of participants: depression only (scored high on an 8-item Mood and Anxiety Symptom Questionnaire (MASQ) anhedonic depression measure and low on anxiety measures), combined (scored high on Penn State Worry Questionnaire (PSWQ) and MASQ measures of anxiety and high on an 8-item MASQ anhedonic depression measure), or control (scored low on anxiety and depression measures. The control group was included in order to investigate whether patterns of preferential attentional processing of unpleasant or emotionally arousing stimuli were specific to the depression only or combined groups. Pleasant and unpleasant stimuli were matched on emotional arousal level.

Critical differences in the nature and timing of attention to emotion were explored in the three groups. (1) It was unclear whether early effects would be present in the depression only or combined group, given inconsistency in the literature of early effects in depression and a general lack of consideration of co-occurring anxiety. If attentional bias is relatively automatic and specific to unpleasant stimuli in depression with and without anxiety, then P100 and/or posterior visual N200 amplitude should be larger for unpleasant than pleasant words. Alternatively, if initial bias is more broadly associated with emotional arousal, then P100 and/or posterior visual N200 amplitude should be larger for both unpleasant and pleasant than neutral words. (2) Later effects were predicted to occur in depression with and without co-occurring anxiety, given a literature documenting later effects in both depression and anxiety. If later, more strategic processing is specific to unpleasant stimuli in depression with and without co-occurring anxiety, then P300 amplitude should be larger for unpleasant than pleasant words. Alternatively, if later, more strategic processing is more broadly associated with emotional arousal, then P300 amplitude should be larger for both unpleasant and pleasant than neutral words. (3) If unpleasant or emotionally arousing words are attended followed by avoidance, than P100 and/or posterior visual N200 amplitude should be larger and P300 amplitude smaller for unpleasant or emotionally arousing stimuli in depression with and without co-occurring anxiety. (4) Gender may moderate early or later attentional processing and was included as an exploratory variable in present analyses. (5) P100, posterior visual N200, and P300 effects may be more pronounced over right posterior regions in the combined group and less pronounced in the depressed group, consistent with previous research regarding regional EEG, ERP, and fMRI patterns in depression with and without co-occurring anxiety (e.g., Heller and Nitschke, 1998; Keller et al., 2000; Sumich et al., 2006; Engels et al., 2010).

## MATERIALS AND METHODS

Much of the methods section, including stimuli and experimental design, EEG recording procedure, and data reduction and analysis procedures overlap with Sass et al. (2010) and to some extent with Fisher et al. (2010) and Stewart et al. (2010). Method details are included here in slightly modified (not verbatim) form from Sass et al. (2010).

All participants provided informed consent, and all procedures were approved for ethical considerations by the University of Illinois Institutional Review Board. A total of 4,457 college undergraduates were screened for the study. Participants were 92 (49 female) paid volunteers (mean age = 19.0, SD = 1.9) recruited via group questionnaire screening sessions^1^,^2^. Participants were 82% European American and were classified as high anhedonic depression (*n* = 24; 11 female), combined (*n* = 27; 19 female), or control (*n* = 41; 19 female) on the basis of responses on the PSWQ and MASQ. Compared to the total sample screened for the study, the anhedonic depression group scored at or above the 80th percentile on an eight-item depressed-mood subscale (Nitschke et al., 2001) of the MASQ Anhedonic Depression scale, shown to predict diagnostic category membership (Bredemeier et al., 2010). The anhedonic depression group also scored at or below the 50th percentile on the PSWQ and on the MASQ Anxious Arousal scale. The combined group scored at or above the 80th percentile on all three scales. The control group scored at or below the 50th percentile on all three scales. **Table 1** presents the means and standard deviations of the groups for the questionnaire measures.

The Structured Clinical Interview for Axis I Disorders, Non-Patient Edition (First et al., 1997), was administered to all participants to assess to what degree selecting participants based on the questionnaire measures yielded significant Axis I disorders. Participants were not selected based on DSM diagnosis, because DSM-defined anxiety and depression disorders include (to varying degrees) anxious apprehension, anxious arousal, and anhedonic depression. Selecting participants based on DSM category would likely result in missed sensitivity in uncovering brain mechanisms (e.g., see Engels et al., 2007, 2010; Herrington et al., 2010) distinctly associated with attentional processing as a function of the transdiagnostic dimensions of anhedonic depression and anhedonic depression co-occurring with anxious arousal and anxious apprehension.

Lifetime DSM-IV-TR (American Psychiatric Association, 2000) diagnoses were determined by a trained clinical psychology doctoral student interviewer and reviewed by a consensus team consisting of a second trained clinical psychology doctoral student interviewer and a clinical faculty supervisor (Gregory A. Miller). Although participants were not selected based on DSM-IV-TR depression or anxiety disorder diagnosis, approximately 25% of the individuals in the depression only and 59% of the combined group met criteria for a lifetime history of major depressive disorder (MDD) and/or an anxiety disorder. Specifically, of the 24 individuals in the depressed group, six met full criteria for a lifetime history of MDD. Of the 27 individuals in the combined group, 11 had a lifetime history of MDD (three were in a current major depressive episode) and 13 had a lifetime history of an anxiety disorder (anxiety disorder not otherwise specified, generalized anxiety disorder, obsessive compulsive disorder, posttraumatic stress disorder, social phobia). Control participants did not meet criteria for any lifetime DSM-IV-TR disorder. Therefore, the questionnaire measures used to screen individuals for the present combined and depressed groups provided a substantial number of participants meeting criteria for DSM-IV-TR diagnoses of MDD and/or an anxiety disorder.

The groups did not differ in age. All participants were determined to be right-handed by the Edinburgh Handedness Inventory (Oldfield, 1971), had self-reported normal color vision, and were native speakers of English. Participants were informed of the procedures of the study and given a laboratory tour. Exclusion criteria included DSM-IV-TR alcohol or drug abuse or dependence within the past 3 months, experience with electroshock therapy, multiple sclerosis, epilepsy, current pregnancy, claustrophobia, moderate to severe head injury, loss of consciousness for 10 min or more, and contraindications for MRI participation (including metal present in the body).

### STIMULI AND EXPERIMENTAL DESIGN

STIM software (James Long Company, Caroga Lake, NY, USA) controlled word presentation and button-press response recording. Neutral blocks were interleaved between blocks of pleasant and unpleasant emotion words. Two hundred fifty-six words were delivered to participants in 16 blocks (four pleasant, eight neutral, four unpleasant) of 16 trials. A word was presented in the center of the computer screen for 1500 ms at the beginning of each trial, followed by a fixation cross for 275 to 725 ms (onset-to-onset ITI 2000 +/- 225 ms). Each trial consisted of a single word presented in one of four colors (red, yellow, green, blue) on a black background. Each color appeared equally often with each word type (pleasant, neutral, unpleasant). Participants completed an emotion-word Stroop task in both EEG and fMRI sessions that were counterbalanced to precede each other equally often. The present report is based on the EEG data. Participants were randomly assigned one of eight possible orders. In half of the presentation orders, the first and third blocks were neutral words, and the second and fourth blocks were pleasant and unpleasant, with valence order counterbalanced across participants. In the remaining half of the presentation orders the first and third blocks were either pleasant or unpleasant emotion words and the second and fourth blocks were neutral words. These eight presentation orders were designed to avoid order effects by ensuring that the neutral and emotional words preceded each other equally often. A given word was presented only once per session to control stimulus familiarity. Each color appeared four times within a block and no more than two trials featuring the same color appeared in a row. A brief rest period occurred after every fourth block. In addition to 16 word blocks, four fixation blocks were presented: one at the beginning, one at the end, and two in the middle of the experiment. Specifically, a bright fixation cross was presented for 1500 ms instead of a word, followed by a dimmer fixation cross that always followed word stimuli.

Sixty-four pleasant, 64 unpleasant, and two sets of 64 neutral words were carefully selected from the Affective Norms for English Words set (ANEW; Bradley and Lang, 1999) on the basis of norms for valence, arousal, and frequency of usage in the English language (Bradley and Lang, 1999). Pleasant and unpleasant words were chosen to be high in arousal (arousal mean = 6.53 for pleasant, 6.56 for unpleasant, and 3.81 for neutral stimuli). Words ranged from three to eight letters in length and were presented in capital letters using Tahoma 72-point font. The visual display was 1.35 m from the participant’s eyes for a vertical span of 1.5° and a horizontal span of 2.5–9.3°. The average luminance values of the words presented in red, yellow, green, or blue were 15, 72, 45, and 20 lux, respectively. Verbatim instructions were read by experimenters to ensure consistency. Each participant performed 32 practice trials before the actual task began. There were four buttons on the response box, with each button corresponding to a color. The left middle and index fingers were used to indicate red and green, respectively. The right middle and index fingers were used to indicate yellow and blue, respectively. All participants understood task instructions and the mapping between colors and buttons after the practice trials were completed.

### EEG RECORDINGS

Participants were seated in a quiet room that was connected via intercom to an adjacent room where the experimenter controlled EEG data collection and stimulus presentation. A custom Falk Minow 64-channel cap with equidistantly spaced Ag/AgCl electrodes was used to record EEG. The left mastoid was the online reference for all EEG and electrooculogram (EOG) sites. Vertical and horizontal EOG was recorded with electrodes placed above and below each eye and near the outer canthus of each eye for off-line eye-movement artifact correction of EEG. Electrode impedances were below 20 Kohms. Data were digitized at 250 Hz, and half-power amplifier bandpass was 0.1–100 Hz. A Zebris ELPOS digitizer recorded electrode positions (Zebris Medizintechnik, Tübingen, Germany).

### DATA REDUCTION

Muscle, movement, and other artifacts were removed manually. Eye blinks were corrected using Brain Electrical Source Analysis (BESA 5.1.8) software (Berg and Scherg, 1994). If a channel was off-scale for more than 10% of trials, all trials for that channel for a given subject were removed from analyses. All channels for epochs in which a single channel was off-scale were discarded. Artifact correction did not differentially affect the number of remaining pleasant, neutral, and unpleasant trials, Emotion *F*(2,89) = 1.45, *p* = 0.237^3^, and did not differ by group or gender. Only correct trials were averaged for the pleasant, neutral, and unpleasant conditions. The electrode configuration was transformed using spherical spline interpolation to BESA’s standard 81-channel montage (Perrin et al., 1989), reflecting the 10–10 system. An average reference (the mean voltage over the 81 standard virtual scalp electrodes) was computed for each time point, and data were exported from BESA. Each channel was baseline-adjusted in custom Matlab software by subtracting the average amplitude for the 200 ms before stimulus onset.

Three ERP components were scored: P100 (88–128 ms), N200 (160–240 ms), and P300 (448–580 ms). A 101-weight, 0.1–20 Hz digital filter was used for P100 and N200, and a 101-weight, 0.1–8 Hz digital filter was used for P300 (Cook and Miller, 1992; Nitschke et al., 1998; Edgar et al., 2005). A combination peak/area measure was used to avoid spurious peaks driving amplitude measures. Voltage 48 ms around the peak was averaged for the early (P100, N200) components, and voltage 96 ms around the peak was averaged for the late (P300) component. Latency associated with each peak was also recorded.

Sites for P100 and N200 were chosen based on examination of current source density (CSD) estimates across conditions and groups. CSD estimates were used as an estimate of the contribution of the immediately underlying cortical surface to the recorded electrode signal (Nunez et al., 1999). Voltage associated with amplitude values at sites where CSD activity was maximal for P100 (P7, P8, PO7, PO8, O1, O2) and N200 (P7, P8, P9, P10, PO7, PO8, PO9, PO10) were averaged together by hemisphere for these bilaterally distributed components. Voltage associated with amplitude values at sites for P300 (P1, P2, P3, P4, CP1, CP2, CP3, CP4) were averaged by hemisphere. Sites for P300 were chosen based on previous emotion-word Stroop studies (e.g., van Hooff et al., 2008; Sass et al., 2010) and inspection of the grand-average waveforms where effects were maximal.

## RESULTS

### BEHAVIORAL PERFORMANCE

Pleasant, neutral, and unpleasant RT was analyzed for correct trial responses between 350 and 1400 ms (*M* = 671 ms, SD = 106 ms). 4.5% of RT data were lost due to the RT criterion of < 350 ms or > 1400 ms. Performance accuracy was high (mean number of errors = 4.0, SD = 4.1, of 256 trials). Participants were excluded from EEG analyses if they were excluded from RT analyses, and from RT analyses if they were excluded from EEG analyses. A Group (depression only, combined, control) x Gender (female, male) x Emotion (pleasant, neutral, unpleasant) multivariate analysis of variance (MANOVA) was conducted. Levels of the emotion factor were ordered pleasant, neutral, and unpleasant in order to take advantage of *a priori* orthogonal linear (valence: comparing pleasant with unpleasant) and quadratic (arousal: comparing pleasant and unpleasant with neutral) univariate trends on the emotion factor. All tests were 2-tailed using an alpha level of 0.05 and *p*-values reflect the Huynh–Feldt correction for sphericity where appropriate. No main effects or interactions were significant for RT.

### EARLY EMOTION-WORD PROCESSING

**FIGURE 1:**
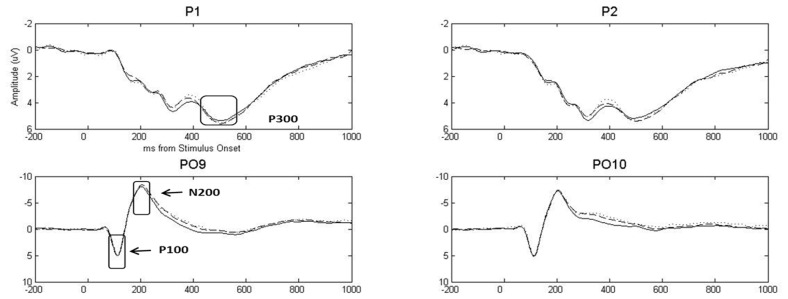
**Grand-average event-related potential waveforms for representative posterior sensors, highlighting P100, N200, and P300.** Dotted, solid, and dashed lines represent pleasant, neutral, and unpleasant stimuli, respectively. Stimulus onset was at time = 0 ms.

A Group (depression only, combined, control) x Gender (female, male) x Emotion (pleasant, neutral, unpleasant) x Hemisphere (left, right) MANOVA including linear and quadratic trends (described above) was conducted separately for P100 and N200 (see **Figure 1** for grand-average waveforms for representative channels). Reported *p*-values reflect the Huynh–Feldt correction for sphericity where appropriate.

#### P100 amplitude

**FIGURE 2:**
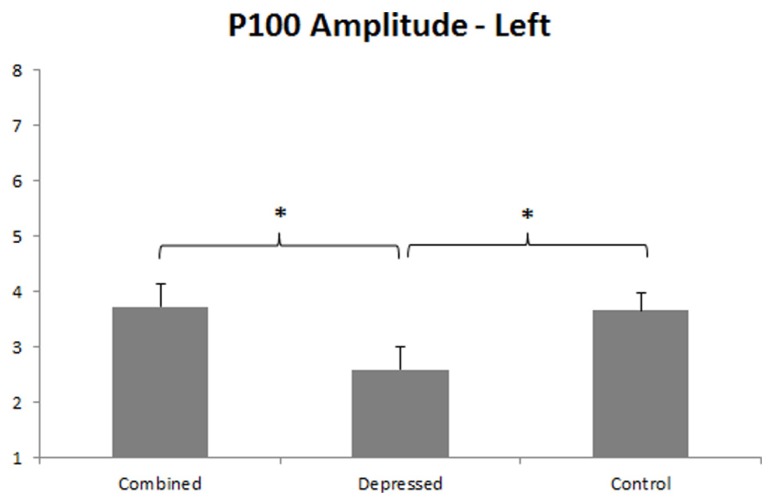
**P100 amplitude in left hemisphere.** Error bars represent 1 SE. Asterisks indicate significant differences (*p* < 0.05), with reduced P100 amplitude in the depressed compared to the combined group, and in the depressed compared to the control group.

**FIGURE 3:**
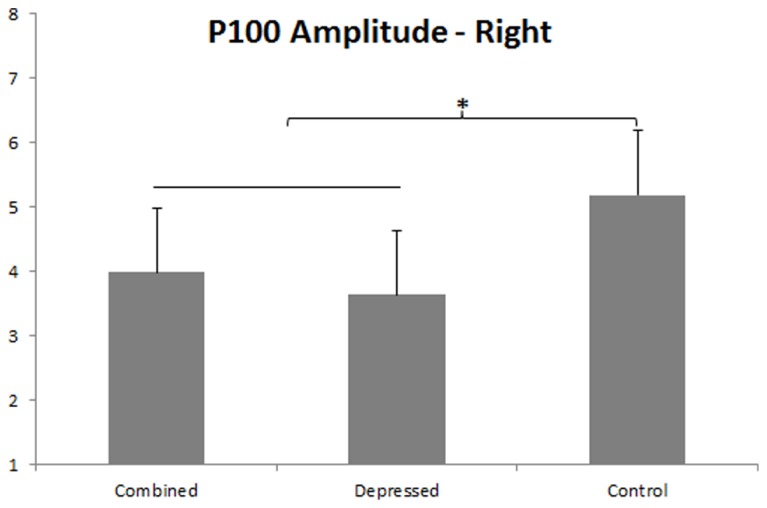
**P100 amplitude effect in right hemisphere.** Error bars represent 1 SE. The asterisk indicates (*p* < 0.05), that both depressed groups showed reduced P100 amplitude compared to the control group.

P100 was larger over right than left hemisphere, *F*(1,86) = 19.67, *p* < 0.001, and a Group main effect, *F*(2,86) = 3.93, *p* = 0.023, was qualified by a Group x Hemisphere interaction, *F*(2,86) = 3.09, *p* = 0.050. Separate Group ANOVAs were done for each hemisphere. The Group effect was significant over left, *F*(2,89) = 3.34, *p* = 0.040, and right, *F*(2,89) = 4.29, *p* = 0.017, hemispheres, dissected with orthogonal Group contrasts for each hemisphere. The first contrast compared the combined with the depressed group, and the second contrast pooled depressed groups and compared them with controls. P100 amplitude was smaller over left hemisphere in the depressed than combined group, *p* = 0.015, and the combined group did not differ from controls (see **Figure 2**). P100 amplitude was smaller over right hemisphere in both depressed groups compared to controls, *p* = 0.005, and the depressed and combined group did not differ from one another (see **Figure 3**).

#### N200 amplitude

**FIGURE 4:**
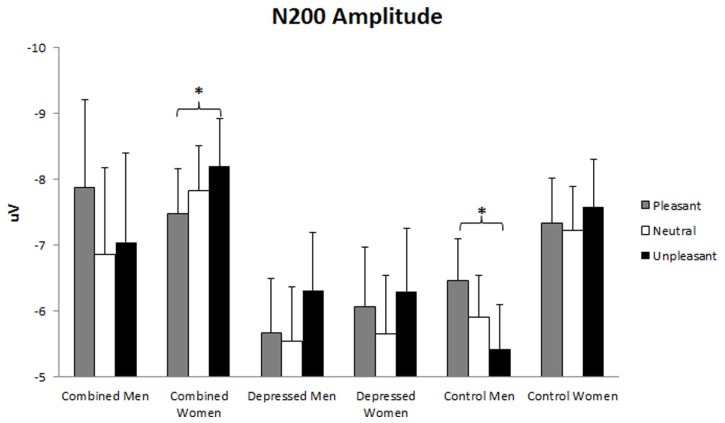
**N200 amplitude.** Error bars represent 1 SE. N200 amplitude valence effect in combined women and control men. The asterisks represent significant differences with *p* < 0.05 in combined women (unpleasant > pleasant) and control men (pleasant > unpleasant).

N200 amplitude was larger over left than right hemisphere, *F*(1,86) = 7.59, *p* = 0.007. A main effect of Emotion, *F*(2,86) = 3.52, *p* = 0.032, was evaluated with linear and quadratic contrasts. N200 amplitude was larger for emotionally arousing than neutral words, *F*(1,86) = 11.43, *p* = 0.010. In addition, a Gender x Emotion effect, *F*(2,86) = 3.40, *p* = 0.036, was qualified by a Group x Gender x Emotion trend, *F*(4,86) = 2.32, *p* = 0.059. This latter interaction was dissected with Gender x Emotion ANOVA analyses for each group, following hypotheses 1, 3, and 4. A Gender x Emotion interaction was present in the combined, *F*(2,25) = 3.83, *p* = 0.028, and control, *F*(2,39) = 4.51, *p* = 0.013, but not depressed group. This interaction was dissected with separate Emotion ANOVAs for each gender within the combined and control groups using linear and quadratic contrasts. In the combined group, N200 amplitude was larger for unpleasant than pleasant words in women but not men, linear *F*(1,18) = 5.00, *p* = 0.038. In the control group, N200 amplitude was larger for pleasant than unpleasant words in men but not women, linear *F*(1,21) = 9.65, *p* = 0.005 (see **Figure 4**).

### LATER EMOTION-WORD PROCESSING

#### P300 amplitude

**FIGURE 5:**
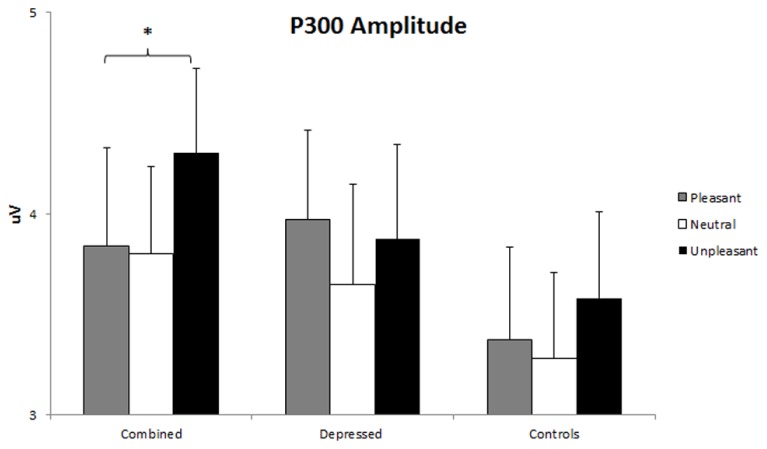
**P300 amplitude over right hemisphere.** Error bars represent 1 SE. P300 amplitude valence effect (unpleasant > pleasant) over right hemisphere sensors (P1, P3, CP1, CP3) in the combined group. The asterisk represents *p* < 0.05 in the combined group (unpleasant > pleasant).

A Group x Gender x Emotion x Hemisphere MANOVA including linear and quadratic trends (described above) was conducted for P300 amplitude. An Emotion effect, *F*(2,86) = 3.95, *p* = 0.021, was followed up with linear and quadratic contrasts. As expected, P300 amplitude was larger for pleasant and unpleasant than neutral words, quadratic Emotion *F*(1,86) = 7.22, *p* = 0.009. A Gender x Hemisphere interaction, *F*(1,86) = 8.84, *p* = 0.004, was investigated with separate Hemisphere ANOVAs for each gender. Only men had larger P300 amplitude over the right than left hemisphere, *F*(1,42) = 4.72, *p* = 0.036. A Group x Hemisphere effect, *F*(1,86) = 3.51, *p* = 0.034, was qualified by a Group x Emotion x Hemisphere interaction, *F*(4,86) = 2.44, *p* = 0.049, dissected with separate Emotion x Hemisphere ANOVAs for each group, following hypotheses 2, 3, and 5. A linear Emotion x Hemisphere interaction, *F*(2,26) = 3.32, *p* = 0.044, emerged in the combined group only. This interaction was dissected with separate Emotion ANOVAs for each hemisphere within the combined group using linear and quadratic contrasts on the emotion factor. P300 amplitude was larger for unpleasant than pleasant stimuli over the right but not left hemisphere, linear *F*(1,25) = 4.35, *p* = 0.047 (see **Figure 5**).

## DISCUSSION

Previous literature provides inconsistent evidence regarding the nature and timing of attentional biases in depression, and unexamined co-occurring anxiety may contribute to this inconsistency. The present study investigated the role of co-occurring anxiety and whether early, relatively automatic, or later, less automatic phenomena manifested in the ERP evidence would support attentional bias in favor of unpleasant or emotionally arousing stimuli in depression. Gender was included as an exploratory variable.

Evidence of early biased processing of unpleasant or emotionally arousing stimuli was absent in the depression-only group. The combined group, however, showed evidence of both an early and a later attentional bias in favor of unpleasant information. Posterior N200 amplitude was larger in women with both depression and anxiety symptoms for unpleasant than for pleasant stimuli, presumably reflecting relatively automatic processing of unpleasant words, consistent with other emotion-word Stroop studies finding modulation of an EPN for emotional compared to neutral stimuli (Franken et al., 2009; Sass et al., 2010). That an early effect modulated by emotion was not evident in the depression-only group suggests that high levels of anxiety are necessary to elicit evidence of attentional bias in depression, consistent with findings of Markela-Larenc et al. (2011).

The combined depression and anxiety group also showed evidence of biased processing later in the trial (larger P300 amplitude for unpleasant than pleasant over right hemisphere), reflecting biased processing of unpleasant information at a later, more elaborative stage. This finding is consistent with emotion-word Stroop studies showing P300 amplitude modulation by emotion (e.g., Li et al., 2007; Franken et al., 2009; Sass et al., 2010). This finding is also consistent with studies finding greater right-lateralized posterior brain activity in depression co-occurring with anxiety (e.g., Engels et al., 2010).

The later preferential attention to unpleasant information seen in the combined group was not observed in the depression-only group. The present depression sample was unusual in that participants were selected only if they scored high on a measure of anhedonic depression and low (in the control group range) on two measures of anxiety, allowing the relatively pure influence of anhedonic depression to be examined. Given present evidence of attentional bias effects in the combined but not depressed group, results indicate that attentional bias effects sometimes found in depressed samples may be due to co-occurring anxiety.

The depression-only group produced smaller P100 amplitude over left hemisphere than the combined group, consistent with EEG studies showing less left than right hemisphere activity in depressed compared to anxious individuals. These findings are also consistent with fMRI results revealing reduced left dorsolateral prefrontal cortical activity in depression when co-occurring anxiety is taken into account (Engels et al., 2010; Herrington et al., 2010).

In addition, both depressed groups showed reduced P100 amplitude over right hemisphere compared to controls that was not specific to emotional stimuli. This result suggests that depressed groups’ detection of visual stimuli is generally dampened at this early time point (~100 ms), despite early (~200 ms), and later (~500 ms) ERP effects showing differential detection and processing of unpleasant stimuli in the combined group. This effect is in contrast to early P100 amplitude effects modulated by anxiety in previous emotion-word Stroop studies (Li et al., 2007; Sass et al., 2010), suggesting that high levels of anxiety and lower levels of depression are necessary to elicit early (~100 ms) emotion effects. P100 amplitude was not modulated by emotion in the present study, consistent with a number of previous studies failing to find early emotion effects in depression (e.g., Rossignol et al., 2008; Gotlib and Joormann, 2010).

There are several caveats and limitations associated with the present study. First, RT evidence of attentional bias was not obtained in the present study. RT is the end-stage of a number of different brain processes, and a lack of RT effects despite ERP effects in the present sample indicates biased processing at stages prior to response execution. This pattern of effects is consistent with and informs studies finding small or no RT effect sizes using the emotion-word Stroop task in samples with sub-clinical depression (for review, see Epp et al., 2012) and anxiety (Koven et al., 2003). Second, a larger sample and equal gender distribution would be better suited to examining gender differences in emotional information processing that may exist in the depression-only and combined groups. Third, because the Group x Gender x Emotion N200 amplitude effect was at trend level, this result should be interpreted tentatively pending replication. Fourth, in averaging across conditions for ERP analyses, pleasant and unpleasant trials were averaged separately before pooling them for comparison to neutral using quadratic contrasts. It is possible that the lower number of trials contributing to the pleasant and unpleasant ERP averages (*n* = 64 for each emotion condition) would contribute to higher amplitude scores than for the neutral averages (*n* = 128), due to the possibility of more noise in the averages with fewer trials. This issue is only relevant to comparisons of emotionally arousing with neutral stimuli and not to comparisons of pleasant with unpleasant stimuli (thus not affecting the main findings in the present paper, of greater N200 and P300 amplitude for unpleasant than pleasant stimuli in the combined group). Given that signal-to-noise reduction is a function of a ratio of the square root of the number of trials comprising an average, and 64 is a reasonable number of trials to begin with, the difference in the number of trials contributing to the emotionally arousing and neutral averages should not have much differential impact on noise reduction. Furthermore, present emotional arousal main effects for posterior visual N200 and P300 amplitude are consistent with previous research employing an equal number of pleasant, unpleasant, and neutral trials (e.g., Franken et al., 2009), suggesting that the emotional arousal effects found in the present study are not a function of differing trial numbers. Finally, the use of a block design in the present study is helpful in eliciting more sustained emotion effects as might occur in everyday emotional contexts. A block design may not be optimal in distinguishing early, more automatic processing from later, more strategic processing, as top-down expectancy effects may influence early processing (e.g., see van Hooff et al., 2008, for a similar discussion).

Present results can inform interventions for depression with and without co-occurring anxiety. Computerized attention-training programs have been successful in modifying attentional bias and reducing symptoms of depression and anxiety (for meta-analyses see Hakamata et al., 2010; Hallion and Ruscio, 2011). In a study targeting mild to moderate depression with mild levels of co-occurring anxiety, participants in an attention-training condition showed a greater reduction in depressive symptoms than did participants receiving a control intervention (Wells and Beevers, 2010). Another study found small improvements in symptom severity after computerized training among students showing mild depression symptoms, but symptoms worsened in those with moderate to severe depression (Baert et al., 2010). In this latter study, co-occurring anxiety was in the mild to moderate range, leaving open the question of whether individuals without co-occurring anxiety would show similar effects. Future research should build on these initial studies, targeting both earlier and later attentional biases and systematically examining the role of co-occurring anxiety.

The present study indicates that the nature and time course of attention prompted by emotional stimuli differentiates depression with and without combined anxiety, and both depressed groups from controls. In the absence of bias effects in the depression-only group, the combined group showed evidence of both an early and a later attentional bias in favor of unpleasant information. Co-occurring anxiety therefore appears to be an important factor in inconsistent results in previous studies regarding attentional biases in depression. Present findings support previous recommendations for careful experimental control of co-occurring anxiety and for including gender and hemisphere when investigating behavioral and brain correlates of attentional biases in depression. Systematic examination of these issues can yield insights into cognition-emotion phenomena in depression that may improve understanding of etiology and treatment, providing valuable directions for future research.

## Conflict of Interest Statement

The authors declare that the research was conducted in the absence of any commercial or financial relationships that could be construed as a potential conflict of interest.

## Footnotes

1 Ninety-six participants qualified for the present study. Of these, two were disqualified due to missing RT data, and two were disqualified due to having one or more ERP component scores (P100, N200, or P300 amplitude) > 3 SD from the mean.

2 Participants from the present study overlapped with three previous ERP investigations. Fifty-nine participants in the present sample were included in Stewart et al. (2010). Stewart et al. (2010) investigated attentional biases as a function of approach and withdrawal anger styles and did not focus on depression with and without co-occurring anxiety. That study also focused on different ERP components than the present study, including N100, P200, N200, and N400 amplitude at fronto-central and central sites, not investigated in the present study. The only overlapping ERP component investigated in Stewart et al. (2010) and the present study is P300 amplitude, with different sensors used. Forty-two participants were shared with Fisher et al. (2010), who investigated emotional information processing as a function of perceived emotional intelligence and focused on different ERP components including P100 and P300 amplitude (defined using different temporal scoring windows and different sensors than the present study), and P200 and slow wave (neither used in the present study). Finally, 32 controls from the present study were used in Sass et al. (2010)

3 Because the number of neutral trials was double the number of each emotion condition, this analysis involved dividing the number of neutral trials by two and then comparing the number in each condition after artifact removal.
